# Hydroponic cultivation conditions allowing the reproducible investigation of poplar root suberization and water transport

**DOI:** 10.1186/s13007-021-00831-5

**Published:** 2021-12-15

**Authors:** Paul Grünhofer, Yayu Guo, Ruili Li, Jinxing Lin, Lukas Schreiber

**Affiliations:** 1 Department of Ecophysiology, Institute of Cellular and Molecular Botany, University of Bonn, Kirschallee 1, 53115 Bonn, Germany; 2 Beijing Advanced Innovation Center for Tree Breeding by Molecular Design, Beijing Forestry University, Beijing, 10083 China; 3 College of Biological Science and Technology, Beijing Forestry University, Beijing, 100083 China; 4 Institute of Tree Development and Genome Editing, Beijing Forestry University, Beijing, 100083 China

**Keywords:** Abiotic stress, Cultivation conditions, Casparian bands, Suberin lamellae, Root suberin, Poplar, Pressure chamber, Water transport, Hydroponics, Barley

## Abstract

**Background:**

With increasing joint research cooperation on national and international levels, there is a high need for harmonized and reproducible cultivation conditions and experimental protocols in order to ensure the best comparability and reliability of acquired data. As a result, not only comparisons of findings of different laboratories working with the same species but also of entirely different species would be facilitated. As *Populus* is becoming an increasingly important genus in modern science and agroforestry, the integration of findings with previously gained knowledge of other crop species is of high significance.

**Results:**

To ease and ensure the comparability of investigations of root suberization and water transport, on a high degree of methodological reproducibility, we set up a hydroponics-based experimental pipeline. This includes plant cultivation, root histochemistry, analytical investigation, and root water transport measurement. A 5-week-long hydroponic cultivation period including an optional final week of stress application resulted in a highly consistent poplar root development. The poplar roots were of conical geometry and exhibited a typical Casparian band development with subsequent continuously increasing suberization of the endodermis. Poplar root suberin was composed of the most frequently described suberin substance classes, but also high amounts of benzoic acid derivatives could be identified. Root transport physiology experiments revealed that poplar roots in this developmental stage have a two- to tenfold higher hydrostatic than osmotic hydraulic conductivity. Lastly, the hydroponic cultivation allowed the application of gradually defined osmotic stress conditions illustrating the precise adjustability of hydroponic experiments as well as the previously reported sensitivity of poplar plants to water deficits.

**Conclusions:**

By maintaining a high degree of harmonization, we were able to compare our results to previously published data on root suberization and water transport of barley and other crop species. Regarding hydroponic poplar cultivation, we enabled high reliability, reproducibility, and comparability for future experiments. In contrast to abiotic stress conditions applied during axenic tissue culture cultivation, this experimental pipeline offers great advantages including the growth of roots in the dark, easy access to root systems before, during, and after stress conditions, and the more accurate definition of the developmental stages of the roots.

**Supplementary Information:**

The online version contains supplementary material available at 10.1186/s13007-021-00831-5.

## Background

Researchers from various laboratories are actively investigating stress physiological responses of plants which are often associated with the more and more prominent effects of global warming or human agriculture and agroforestry. These effects include prolonged periods of drought or flooding, an increase of soil salinity, or exposure to heavy metals [1–5]. Scientific joint ventures are frequently undertaken to not only unravel and understand the coping mechanisms of plants, but also to propose potentially helpful breeding technologies, beneficial genetic modifications, or more efficient cultivation approaches for the future. This international scientific cooperation raises the need for reproducible and consistent methodologies, allowing a better integration and comparability of results attained with the same species in different laboratories throughout the world. In addition, increased harmonization of experimental approaches would also allow more reliable comparability of entirely different species.

*Populus*, a plant genus gaining increased importance in science and agroforestry [6–9], is generally known for its rapid growth and high productivity, straightforward vegetative propagation, usability in biofuel production, reforestation, and phytoremediation, as well as efficient breeding and transformation possibilities [10–16]. Many poplar species are described to be capable of quick and uncomplicated vegetative reproduction, which not only allows their axenic tissue culture cultivation [16, 17] but also enables the rooting of matured stem cuttings of plants grown in soil for scientific and economic purposes [8, 18]. Alternatively, rooted plantlets from axenic tissue culture may directly be acclimatized to hydroponic conditions [17, 19], with the disadvantage that the roots have been illuminated during in vitro cultivation and the root age is not precisely defined. Either way, the resulting adventitious roots [20] may then be used to study physiological responses of poplar roots towards exposure to different abiotic stress conditions applied to the hydroponic setups [21–25].

The frequent study of root apoplastic barriers (Casparian bands and suberin lamellae) as plant means to adapt to changing environmental conditions in many crop species [26–33] has already led to the refinement of a set of highly elaborate methodologies which are readily available to be adjusted to the increasingly important poplar root research. However, it has been discussed before that either method on its own is not sufficient to reliably elucidate the complex structures, properties, and interactions of suberized transport barriers in roots [5, 34]. It is especially the combination of protocols for consistent plant cultivation, histochemistry, biochemical investigation, transcriptomics, and final functional studies investigating transport physiology that will enable the best comparability to previously published research [21, 22, 35–47].

Here we describe an experimental pipeline including (i) rooting of poplar plants, (ii) cultivation of adventitious roots in hydroponics avoiding root illumination, (iii) histochemical observation of root anatomy, (iv) analytical investigation of root suberization, and (v) examination of root transport physiology, which facilitates generating reliable, consistent, and reproducible results. This hydroponic set-up allows precise abiotic stress treatments and a variety of subsequent measurements (e.g. pressure probe and pressure chamber experiments, RNA sequencing of clean roots).

## Materials and methods

### Plant material and cultivation conditions

**Fig. 1:**
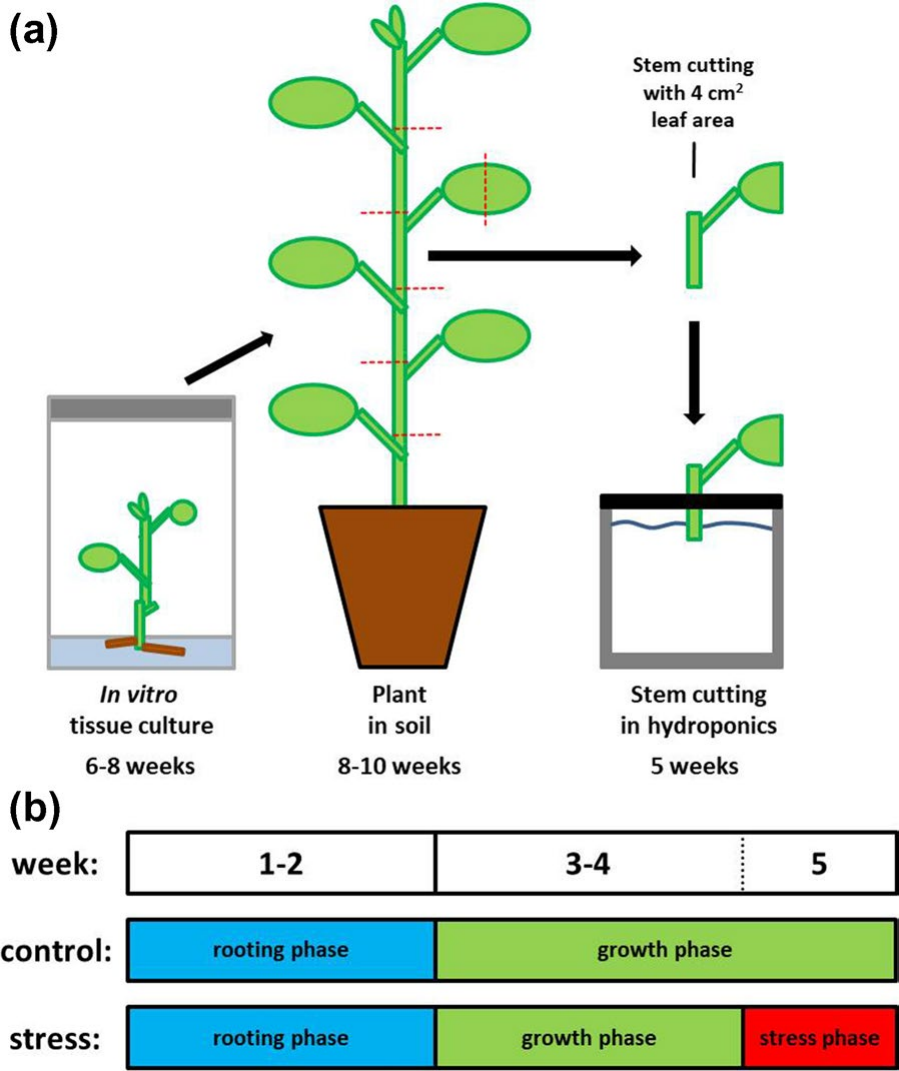
Experimental setup of hydroponic poplar cultivation. Plants cultivated in axenic tissue culture for 6 to 8 weeks were transferred into soil and grown for another 8 to 10 weeks (including 2 weeks of acclimatization with gradually decreased relative humidity) before use in hydroponic experiments (**a**). To initiate a hydroponic experiment, the matured plants were dissected into stem cuttings and fixed to grey, lightproof PVC disks using foam material. The leaf surface area was reduced to 4 cm^2^ to minimize evaporation capacity. During an up to 2-week-long rooting phase in pots filled with non-aerated tap water, rooted stem cuttings were transferred to a new PVC disk located on pots filled with aerated ½ Hoagland solution [49] to grow for further 2 weeks before an optional 1-week-long stress phase in the fifth week (**b**). Five rooted stem cuttings of a single plant yielded one biological replicate. Images in **a** are not to scale

All poplar plants investigated were growing in a climate chamber with controlled long-day (16 h light/8 h dark) conditions (mean temperature of 21(day)/19(night) °C, mean relative humidity of 50(day)/67(night)%, and mean light intensity of approximately 100 µmol m^−2^ s^−1^). For the data presented here, the fully sequenced *P.* × *canescens* (Aiton) Sm. clone “84K” (*P. alba* × *P. tremula* var. *glandulosa*) [48] was investigated. To keep a constant cultivation of poplar plants as stock for follow-up experiments, 84K plants were regularly propagated in axenic tissue culture (½ MS medium, Duchefa Biochemie, Netherlands; 2% saccharose, Carl Roth, Germany; 0.5% Phytagel, Sigma-Aldrich, Germany). To get more detailed information about axenic tissue culture cultivation and micropropagation of commonly investigated poplars, the reader is referred to [17]. For subsequent consistent and reproducible hydroponic experiments, in vitro tissue culture plants were transferred into soil (Einheitserde Classic Type Topf 1.5, Einheitserde Werksverband e.V., Germany) 6 to 8 weeks after propagation, followed by a 2-week-long acclimatization procedure (Fig. 1a). Lids initially covering the plants were gradually lifted within the second week after transplantation and entirely removed at the beginning of the third week. In total, plants were growing in soil for about 8 to 10 weeks before being harvested for hydroponic experiments (Fig. 1a).

**Fig. 2:**
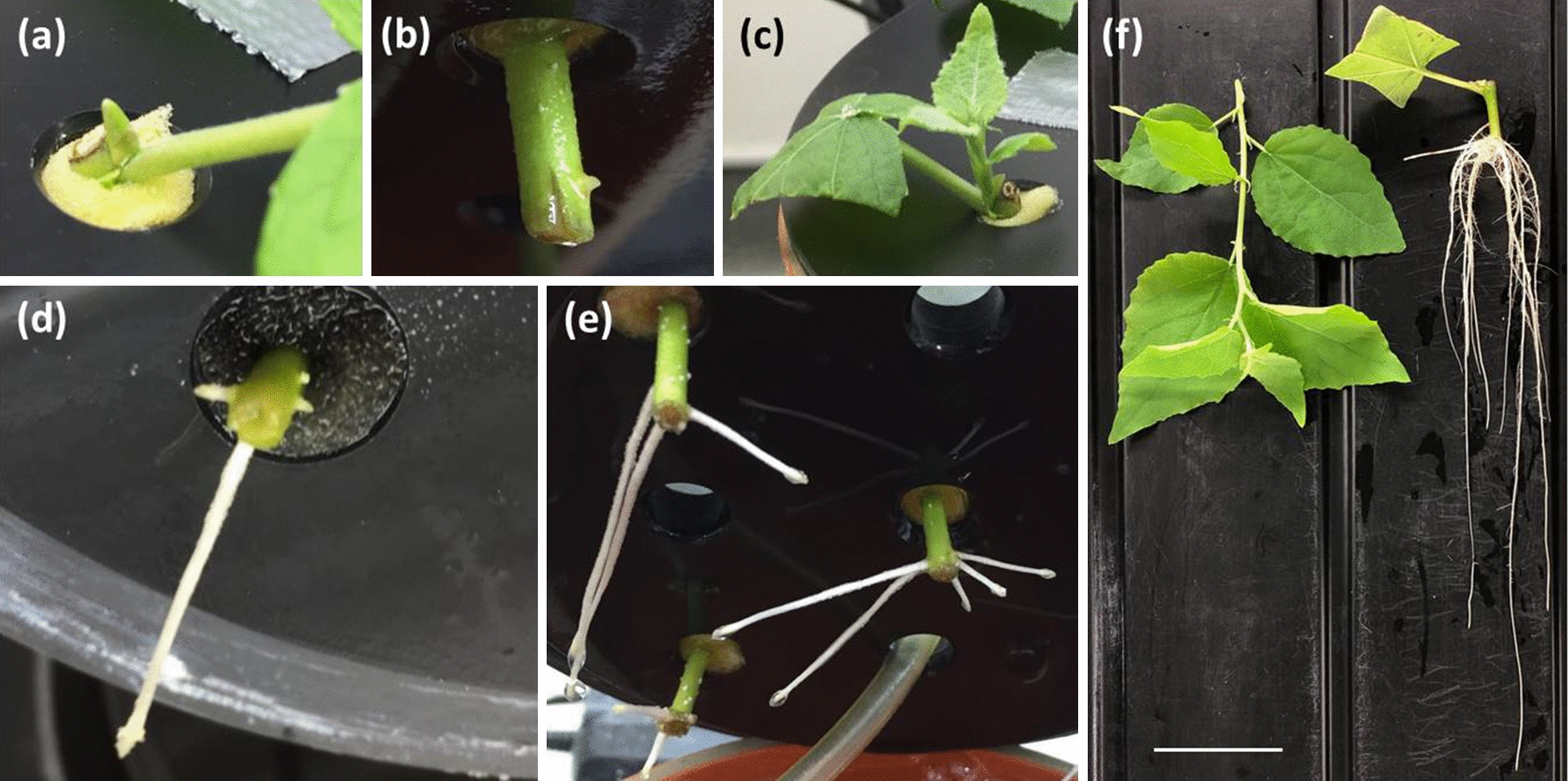
Pictures visualizing poplar plant development in hydroponic cultivation. About 7 days after the start of the rooting phase, the buds began to swell (**a**) and the first roots emerged (**b**). A few days after bud break, fully functional shoots with intact leaves developed (**c**). The highest number of roots per stem cutting was typically achieved within 14 days (**d**) and only a very limited number of roots emerged afterwards (**e**). When plants were harvested after 5 weeks of hydroponic cultivation, the shoots were about 11 cm tall and had eight leaves, whereas eight to ten roots had developed having mean lengths of 13 cm (**f**). Scale bar = 5 cm, only for picture (**f**)

To initiate a hydroponic experiment, 14- to 18-week-old plants (about 50 cm in height and 18 developed leaves) were dissected into phytomers (termed “stem cuttings” hereafter) and the leaf surface area of each stem cutting was reduced to 4 cm^2^ to minimize evaporation capacity (Fig. 1a). Prepared stem cuttings were fixed in grey, lightproof polyvinyl chloride (PVC) disks with holes (1 cm diameter) using foam material. The plastic disks were then placed onto fitting pots (KG pipes DN150 shortened to 20 cm height and sealed with an end cap, resulting in a pot volume of 3.5 L) filled with stagnant tap water to initiate an up to 2-week-long rooting phase (Fig. 1b). The stem cutting development was monitored daily during these 2 weeks. After about 7 days, the first adventitious roots began to emerge (Fig. 2b, Additional file 1: Fig. S1) and rooted stem cuttings were immediately transferred to new PVC disks. This time, the PVC disk was put on a pot filled with aerated ½ Hoagland solution [49] to ensure a sufficient supply of oxygen as well as micro- and macronutrients. Aeration was achieved using aquarium pumps (EHEIM 200, EHEIM GmbH, Germany). Typically after 9 to 11 days, five stem cuttings of a single plant were rooted and combined in one pot to yield one biological replicate. The nutrient solution was exchanged weekly and 4 weeks after the hydroponic setups were started, a stress phase could be initiated (Fig. 1b). All full-scale hydroponic experiments, including the untreated controls as the main objective of this study, were harvested after 5 weeks (Fig. 1b). In addition, some hydroponic setups were harvested earlier after 3 and 4 weeks. Chlorophyll contents (Force A device, Dualex Scientific, France) and stomatal conductances (SC-1 Leaf Porometer, Decagon, USA) of intact leaves, osmotic potentials of leaves, roots, and xylem sap (measured with a freezing point osmometer, OSMOMAT 030, gonotec, Germany), shoot lengths, projected leaf surface area, and root lengths were measured, and roots were kept in fixation solution (3.7% v/v formaldehyde, 10 mM Na_2_HPO_4_, 137 mM NaCl, 2.7 mM KCl, pH 7.4) until further use.

Due to a high variation in rooting efficiency and shoot and root development, the overall root length mean of a given biological replicate (one pot with five stem cuttings) was calculated (typically between 10.6 to 15.1 cm for the 10 replicates investigated here), and roots whose length were close (± 3 cm) to the calculated mean were pre-selected for further experiments to reduce biological variability. In the following histochemical and analytical experiments, the presence of functional anatomical features (root zone only with Casparian bands, root zone with beginning of suberization, root zone of full suberization) was identified, and this developmental pattern of suberization along the root axis was expressed in percent of the whole root length. It was previously shown for barley (*Hordeum vulgare*) that roots of similar age but different lengths due to different cultivation conditions (control vs. osmotic stress) could best be compared by expressing the root development in percentage values [40]. Relative examination also allows the comparison between different plant species (see Fig. 8). This method requires a careful mapping of the root anatomy over the root length. In addition, it holds as long as the stress duration is not too long (not more than a week) so that the growth rate is not changed over prolonged periods of time and the identified root zones have the same functional and developmental stage [40, 43].

### Histochemical observation of Casparian bands and suberin lamellae in poplar roots

Whole adventitious roots with lengths close to the calculated mean were manually divided into 1 cm increments and cross-sectioned with a cryostat microtome (Microm HM 500 M, Microm International GmbH, Germany). To detect Casparian bands, the 30 µm cross-sections were stained with 0.1% (w/v) berberine hemi-sulphate for 1 h and subsequently counterstained with 0.5% (w/v) aniline blue for 30 min [50]. Alternatively, to detect suberin lamellae, the 30 µm cross-sections were stained with 0.01% (w/v) fluorol yellow 088 for 1 h [51]. Epifluorescence microscopy using an ultraviolet (UV) filter set (excitation filter BP 365, dichroic mirror FT 395, barrier filter LP 397; Zeiss, Germany) was carried out and photographs were taken with a Canon EOS 600D camera (Canon Inc., Japan) at ISO 400, 1 s aperture opening, and a 100-fold magnification.

### Analytical investigation of poplar root suberin

To yield one replicate for analytical investigation, 10 to 20 adventitious roots of a hydroponic pot with lengths close around the calculated mean were pooled and dissected into two zones (zone A, no suberization, 0–27.5%; and zone B, patchy suberization, 27.5–100% of the root length), which were defined based on the previous histochemical analyses of roots grown in control conditions (Fig. 4c). This classification into functional developmental zones enables high comparability to earlier studies on barley [5, 40, 52], but also other monocotyledonous [26] as well as dicotyledonous [27, 33] crop species.

The pooled root segments were enzymatically digested for 2 weeks using 0.5% (w/v) cellulase and 0.5% (w/v) pectinase [53]. This included replacement of the digestion solution every 3 days and vacuum infiltration to achieve higher efficiency. To extract soluble lipids, the root segments were exposed to borate buffer for 1 day and were subsequently extracted with 1:1 (v/v) chloroform:methanol for further 2 weeks, again replacing the solution every 3 days. To check for chemical constituents, this typically discarded chloroform:methanol supernatant was also once analyzed by the same following analytical procedure. Dried samples (on polytetrafluoroethylene over activated silica gel) were transesterified with BF_3_-methanol [54] and 10 µg of an internal standard (Dotriacontane, Fluka, Germany) was added to the released suberin monomers. After repeated extraction with chloroform, the entire volume was evaporated under a gentle stream of nitrogen at 60 °C. After adding 150 µl of chloroform, 20 µl pyridine (Sigma Aldrich, Germany), and 20 µl BSTFA (*N*,*O*-Bis(trimethylsilyl)trifluoroacetamide, Macherey–Nagel, Germany), the samples were derivatized for 45 min at 60 °C to mask reactive alcohol and acid groups by trimethylsilyl (TMS) protective groups. Finally, 1 µl of each sample was analyzed by splitless gas chromatography, which was either combined with flame ionization detection (GC-FID: 6890N, Agilent Technologies, USA) or mass spectrometry (GC–MS: 7890B-5977A, Agilent Technology, USA).

The GC-FID system was used for quantitative evaluation, whereas the GC–MS system was used for qualitative identification of suberin monomers using an in-house created library. The temperature program after sample injection was 50 °C for 1 min, a temperature increase of 25 °C min^−1^ up to 200 °C, 1 min at 200 °C, 10 °C min^−1^ up to 320 °C, and a final hold for 8 min at 320 °C [55] and DB-1 columns (30 m length, 0.32 mm diameter, 0.2 µm coating thickness; Agilent J&W) were used. Since no formation of an exodermis (neither Casparian bands nor deposited suberin lamellae) could be observed in the poplar roots, the results of the chemical analysis are representative of the endodermal suberization. To refer the suberin amounts identified to the endodermal surface area (A_en_) of each zone, a truncated cone shape due to growth in root thickness (Fig. 4a) was used for calculation: A_en_ = π (R + r √((R—r)^2^ + h^2^) (R, endodermis radius at basal side of root zone; r, endodermis radius at apical side of root zone; h, length of the individual root zone; r and R were estimated based on the root diameter means of 12 investigated roots grown for 5 weeks in control conditions). In total, ten biological replicates of the control conditions were analyzed.

### Root transport physiology

To analyze the transport physiological properties of whole individual roots cultivated in control conditions, the root pressure probe (thoroughly explained in [39, 56]) was used. To obtain whole adventitious roots with a basal root zone (approximately 20 mm of length) being devoid of lateral roots, basal lateral roots were shaved 7 days prior to measurements using a razor blade according to [57]. After the wounds were healed, this allowed the adventitious roots to be fixed to the root pressure probe using cylindrical silicone seals (Xantopren blue, Heraeus, Germany). The high amount of lignified xylem tissue at the root base (Fig. 4c) greatly facilitated the establishment of a stable root pressure (P_r_), which developed within 30 to 120 min and was around 0.01 to 0.07 MPa.

To briefly explain pressure probe experiments, hydrostatic pressure changes were induced by moving the micrometer screw, resulting in radial water flow into or out of the root. The half-times of water exchange (*t*^*w*^_½_, in s) were used to calculate the hydrostatic hydraulic conductivity (in m s^−1^ MPa^−1^) Lp_r_(HY) = ln(2) (*t*^*w*^_½_ A_r_ β)^−1^. Here, β (MPa m^−3^) denotes the elastic coefficient of the measuring system and A_r_ (m^2^) is the conductive surface area of the root, which was calculated based on a truncated cone shape (see the calculation of A_en_ above). The non-conductive root tip (approx. 10 mm) having no or non-functional xylem vessels as well as the 10 mm basal root segment fixed to the root pressure probe were not included in the calculation.

In contrast, osmotic pressure changes were induced by rapidly exchanging the ½ Hoagland nutrient solution (15 mOsmol kg^−1^) with 5 × concentrated ½ Hoagland nutrient solution (75 mOsmol kg^−1^). To avoid the effects of unstirred layers [39], the nutrient solution was constantly stirred using aeration. In this experiment, the osmotic hydraulic conductivity Lp_r_(OS) was calculated based on the half-time of the (first) water phase (*t*^*w*^_½_) of the biphasic osmotic root pressure relaxation. The reflection coefficient (σ_sr_) of the root for the nutrient ions was calculated using the equation σ_sr_ = ΔP_r_ Δπ^−1^ exp(ln(2) *t*^*s*^_½_^−1^ *t*_min_). Here, ΔP_r_ is the maximum change in root pressure which is caused by the change of the osmotic pressure of the medium (Δπ; π = *R T C*, with *R* being the universal gas constant, *T* being the absolute temperature, and *C* being the osmolarity of the medium). *t*^*s*^_½_ is the half-time of the (second) solute phase of the biphasic osmotic root pressure relaxation and *t*_min_ is the time required to reach minimum root pressure. In total, 16 whole adventitious roots cultivated in control conditions were analyzed for measuring the individual reflection coefficients, of which a subset of 6 roots (being 16.5 cm on average) were also analyzed for both their osmotic and hydrostatic hydraulic conductivity.

The hydraulic properties of the whole root system can also be estimated by measuring the rate of xylem sap exudation in the absence (osmotic, OS) and the presence (hydrostatic, HY) of applied pneumatic pressure. The experiments were conducted according to [37, 58]. The newly grown shoots of 5-week-old stem cuttings were cut off below the first developed leaf (about 10 mm from the stem cutting) underwater using a razor blade to avoid embolisms. The intact root systems of the truncated stem cuttings were then placed in a measuring cylinder filled with the same nutrient solution used for plant cultivation and fixed in a pressure chamber made of steel (Figure 1 in [37]) with previously prepared and longitudinally cut silicone seals (Xantopren blue, Heraeus, Germany) and additional sealing material (Terostat, Germany) for fine adjustment. More than 98% of the root surface area was submerged in the nutrient solution, whereas the base of the stem cutting was located in the air space of the pressure chamber. The remaining petiole of the original leaf with a reduced surface area of 4 cm^2^ was closed using a clamp. This way, only the xylem tissues of the newly developed adventitious roots as well as the xylem of the stem were able to conduct exudates. As soon as air bubbles were observed within the forming exudate droplet (especially with higher applied pneumatic pressures), indicating permeability of the stem cutting to air due to imperfect wounding tissue deposition at the base of the stem cutting, the stem cutting was discarded and not included in the calculations. However, this occurred very rarely.

In the absence of hydrostatic pressure, only the differences between the osmotic pressure (Δπ in MPa) of the nutrient medium and the xylem sap drives the water uptake of the roots, as no more transpirational tension of the truncated shoot is present. Xylem sap exuding from the cut surface was collected in Eppendorf tubes and weighed (assuming a density of 1 to convert weight into volume), allowing the calculation of the volume flow (J_v_ in m^3^ s^−1^) when plotted against the time. The volume flow was then normalized to the exposed root surface area (J_vr_ in m s^−1^), which was determined by scanning after staining the roots in 0.03% (w/v) toluidine blue O (Merck, Germany) for 24 h. The projected root surface areas (trapezoid in shape) were multiplied with π to account for their three-dimensional round geometry. The osmotic potential of the nutrient solution was − 0.037 MPa and that of the exuded xylem sap after growth in control conditions for 5 weeks − 0.123 MPa on average. The reflection coefficient of nutrient ions (σ_sr_) measured with the root pressure probe was 0.47 on average. In combination, this allowed the calculation of the osmotic hydraulic conductivity (Lp_r_(OS) in m s^−1^ MPa^−1^): Lp_r_(OS) = J_vr_ (Δπ σ_sr_)^−1^.

After measurement of the osmotic volume flow, the same root system was used to measure the hydrostatic volume flow by stepwise increasing the applied pneumatic pressure (P in MPa) from 0 to 0.4 MPa above atmospheric with intervals of 0.1 MPa. Exudates were again collected for each applied pressure gradient and the volume flow was estimated. In contrast to the Lp_r_(OS), the hydrostatic hydraulic conductivity (Lp_r_(HY)) was calculated from the slopes of J_vr_ plotted against the applied pneumatic pressure in the linear region of the plots (Fig. 6b) and by considering the combined osmotic and hydrostatic driving forces: Lp_r_(HY) = J_vr_ (P + Δπ σ_sr_)^−1^.

### Stress treatments

To visualize the precision of possible osmotic stress treatments using this hydroponic setup, a series of decreasing water potential levels was performed. The osmotic potential of the nutrient solution was adjusted between − 0.4 and − 1.2 MPa in 0.2 MPa steps following equations in [59] by adding different concentrations of polyethylene glycol (PEG8000, Carl Roth, Germany), a non-toxic polyether frequently used to simulate water deficits. The osmotic potentials were confirmed with a WP4C Dewpoint PontentiaMeter (Decagon Devices, USA). For this demonstration of applicability, only one biological replicate was performed per treatment. To exclude roots that developed newly during the fifth week and have not been exposed to the osmotic stress conditions entirely, only roots that were longer than 5 cm were considered in the evaluation.

### Statistical analysis

The data analysis of the stress experiments was carried out using OriginPro 20 (OriginLab Corporation, USA). As no normal distribution of the root lengths was found (Shapiro–Wilk test), a nonparametric Kruskal–Wallis ANOVA (with subsequent Dunn’s test) at a significance level of p ≤ 0.05 was executed. Significant differences are indicated by differential letters. To visualize the data, boxplots or means with standard deviation are shown.

## Results

### Plant development in control conditions

**Fig. 3:**
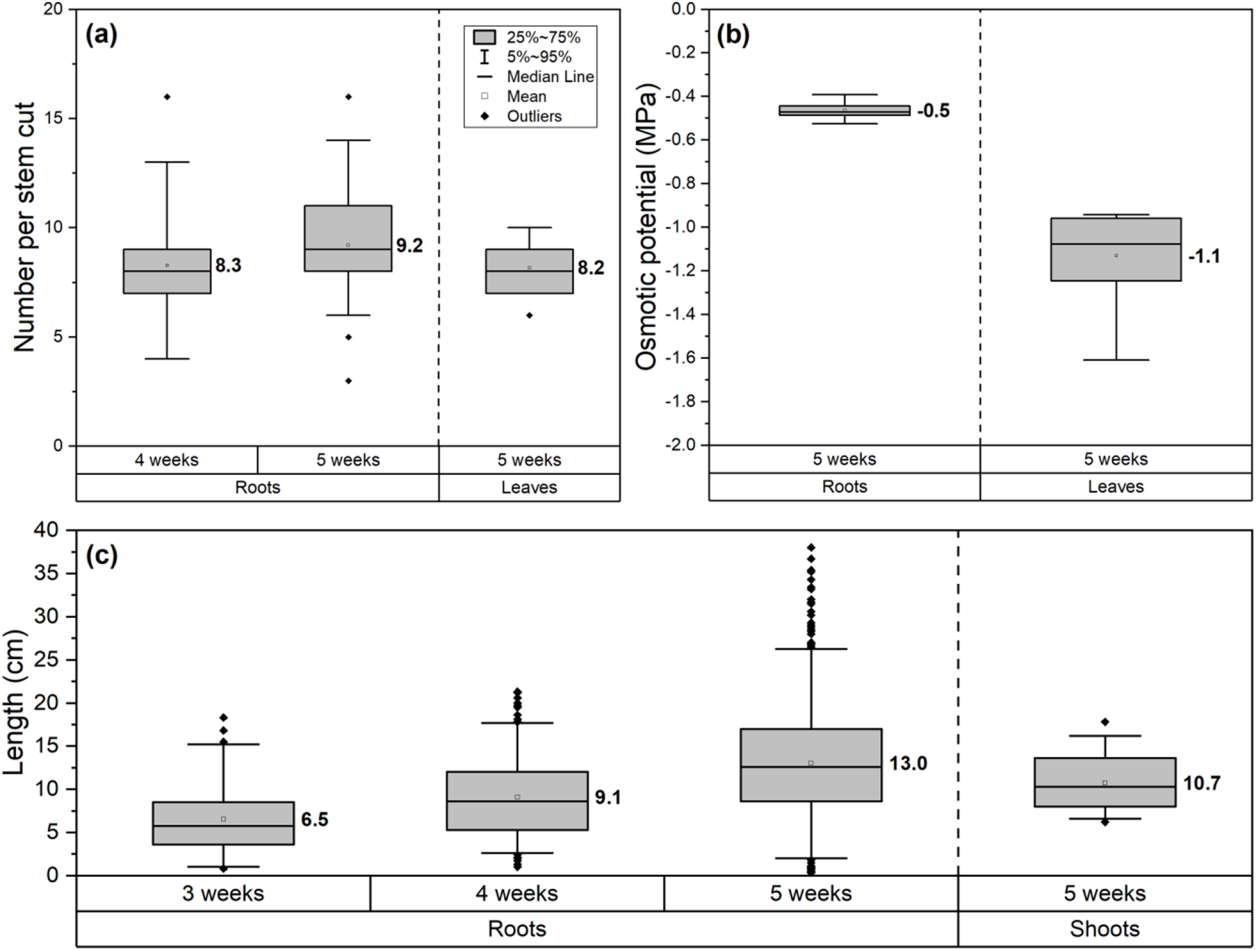
Developmental states of poplar stem cuttings during hydroponic cultivation in control conditions. After new roots and shoots had developed in the first 14 days (rooting phase), only very few additional roots emerged during later periods of the hydroponic cultivation (**a**). After 5 weeks of cultivation, the roots and leaves showed a typical osmotic potential gradient (**b**), and the roots elongated linearly with about 3 cm per week on average (**c**). Boxplots are based on n = 30–50 (**a**) stem cuttings, 7 (**b**) roots and leaves, and 35–512 (**c**) individual roots or shoots from 10 biological replicates

About 7 days after the beginning of a hydroponic experiment, the buds began to swell and the first stem cuttings developed first emerging adventitious roots (Fig. 2a, b, Additional file 1: Fig. S1). During the next days, also the remaining stem cuttings showed emerging roots and after 9 days on average more than 50% of prepared stem cuttings were rooted (Additional file 1: Fig. S1). Latest within 14 days, sufficient stem cuttings had developed roots (Additional file 1: Fig. S1). New root emergence decreased significantly after eight roots were developed per stem cutting on average (typically also within the first 14 days), and only a few delayed roots emerged throughout the remaining experiment (Fig. 3a). All in all, stem cuttings that were taken from the middle fraction of the plant rooted the quickest and most reliable, whereas the first 2 to 4 apical stem cuttings tended to develop no adventitious roots at all. Shortly after bud swelling and first root emergence, also the bud break took place (Fig. 2c, Additional file 1: Fig. S1), and shoots of about 11 cm height with approximately eight leaves developed within 5 weeks (Figs. 2f, 3a, c). The root lengths increased steadily with each week to reach a final average length of 13 cm after 5 weeks (Figs. 2d–f, 3c). When plants cultivated in control conditions were harvested after 5 weeks, they exhibited osmotic potentials of − 0.5 MPa (roots) and − 1.1 MPa (leaves) (Fig. 3b), leaf chlorophyll contents were 14.4 µg cm^−2^, combined projected leaf surface areas were 146.5 cm^2^, and stomatal conductances were 304.7 mmol m^−2^ s^−1^ (Additional file 1: Fig. S2). This experimental setup was also successfully tested with another *P.* × *canescens* clone “INRA 717-1B4” (*P. tremula* × *P. alba*) [60] (no data shown).

### Histochemical observation of Casparian bands and suberin lamellae in poplar roots

**Fig. 4:**
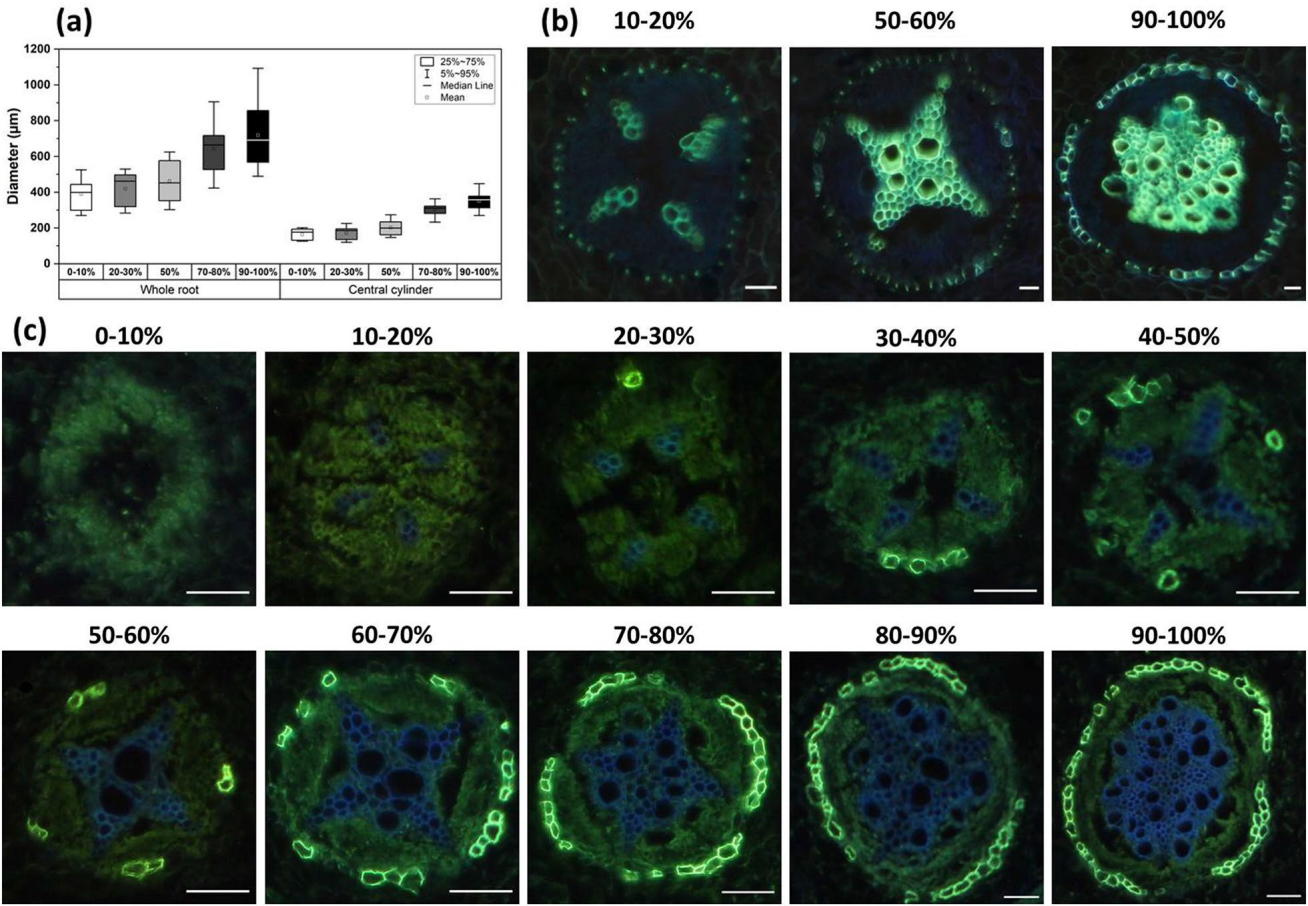
Histochemical analysis of poplar roots cultivated in hydroponic control conditions for 5 weeks. **a** Growth in thickness results in a conical three-dimensional geometry of the whole root as well as the central cylinder (n = 12 roots). **b** Endodermal Casparian band development. Already closely behind the root tip, the first thin Casparian bands were visible in radial endodermal cell walls at 10–20% relative distance. The Casparian bands constantly developed and increased in length even in the basal root segments of 90–100% relative root length. No Casparian bands developed in the hypodermis (no picture shown). **c** Endodermal suberin lamellae development. At 27.5% relative root length on average, first suberized endodermal cells were visible. Suberization increased in a patchy manner adjacent to the phloem poles to reach an almost full suberization in the basal 90–100% relative distance. No suberin lamellae developed in the hypodermis (no picture shown). Scale bars = 50 µm

The histochemical observations revealed the typical primary anatomy of a dicotyledonous root with an increase in diameter from tip to base (Fig. 4a). This resulted in a distinct conical geometry of all adventitious roots. Neither Casparian bands nor suberin lamellae could be identified in the hypodermis under control conditions. In the endodermis, enclosing the central cylinder with tetrarch vascular bundle, Casparian bands were already visible in all radial endodermal cell walls 10–20% behind the root tip (Fig. 4b), which was in close proximity to the onset of xylem development (Fig. 4c). In contrast to the Casparian bands, first suberized endodermal cells could be observed at a 27.5% distance (n = 6 roots) from the root tip on average (Fig. 4c) and thus defining a zone of no suberization (zone A, 0–27.5%). Endodermal suberization constantly increased in a patchy manner adjacent to the phloem poles, to reach an almost full suberization in the last 90–100% of root length (Fig. 4c), resulting in the definition of a second zone B (patchy suberization) of 27.5–100% relative root length. As full suberization has rarely been observed, no third zone C was defined.

### Analytical investigation of poplar root suberin

**Fig. 5:**
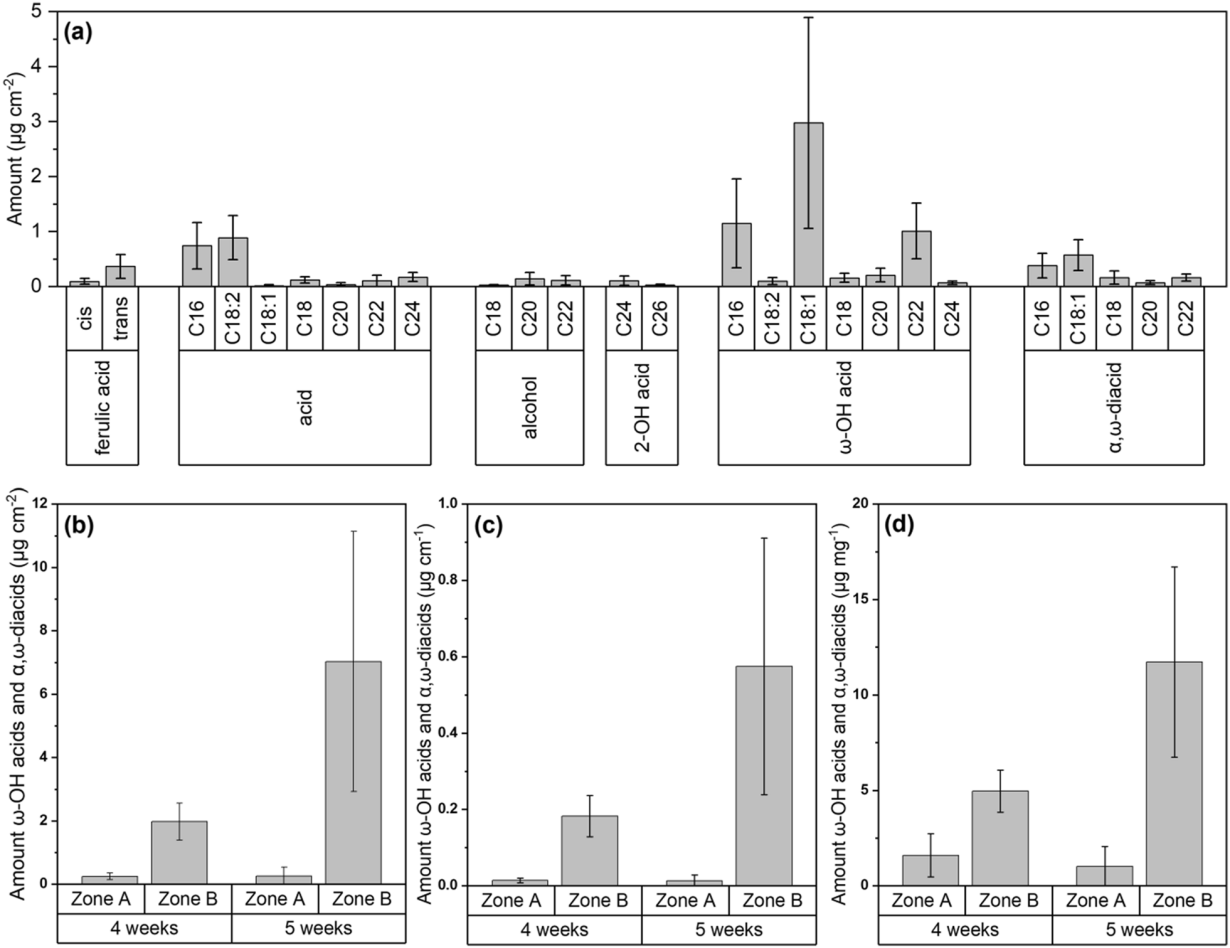
Chemical analysis of suberin in poplar roots. **a** Monomer composition of poplar root suberin harvested after 5 weeks of hydroponic cultivation in control conditions. The representative qualitative and quantitative composition of zone B is given. Means with standard deviations (n = 10 biological replicates) are shown. acid = primary acid, alcohol = primary alcohol, 2-OH acid = 2-hydroxy acid, ω-OH acid = ω-hydroxy acid, α,ω-diacid = α,ω-dicarboxylic acid. **b**–**d** Amounts of suberin diagnostic ω-hydroxy acids and α,ω-dicarboxylic acids in root zone A and B. Roots were harvested after 4 and 5 weeks of hydroponic cultivation in control conditions. Identified amounts were related to endodermal surface area (**b**), root length (**c**), and root dry-weight (**d**). Means with standard deviations (n = 10 biological replicates) are shown

Chemical analysis revealed that suberized cell walls consisted of the aromatic compound ferulic acid and linear long-chain aliphatic compounds with the major substance classes of primary acids, primary alcohols, 2-hydroxy acids, ω-hydroxy acids, and α,ω-dicarboxylic acids of chain-lengths between C16 and C26 (Fig. 5a), as well as further aromatic benzoic acid derivatives (Additional file 1: Fig. S3b, c). In the chloroform:methanol supernatant, obtained from the extraction of isolated suberized cell walls, most prominent compounds were primary acids, primary alcohols, 2-hydroxy acids, benzoic acid derivatives, and phytosterols (Additional file 1: Fig. S3a). Further analysis of suberin amounts was restricted to the two most suberin diagnostic substance classes ω-hydroxy acids and α,ω-dicarboxylic acids (Fig. 5b–d), which were exclusively identified as monomers released from the suberin polymer after transesterification. After 5 weeks of hydroponic cultivation, suberin amounts in roots were 0.3 µg cm^−2^ in zone A and 7.0 µg cm^−2^ in zone B (Fig. 5b). As different varieties of stress may, due to a decently progressed root development, best be introduced after 4 weeks of hydroponics, also the suberin amounts after 4 weeks were investigated. The suberin amounts of zone A had not increased, but the suberin amounts of zone B were 3.5-fold higher after 1 week of further root development (Fig. 5b). No big differences in proportions of suberin amounts could be observed when related to root diameter (Fig. 5b), root length (Fig. 5c), or root dry-weight (Fig. 5d).

### Root transport physiology

**Fig. 6:**
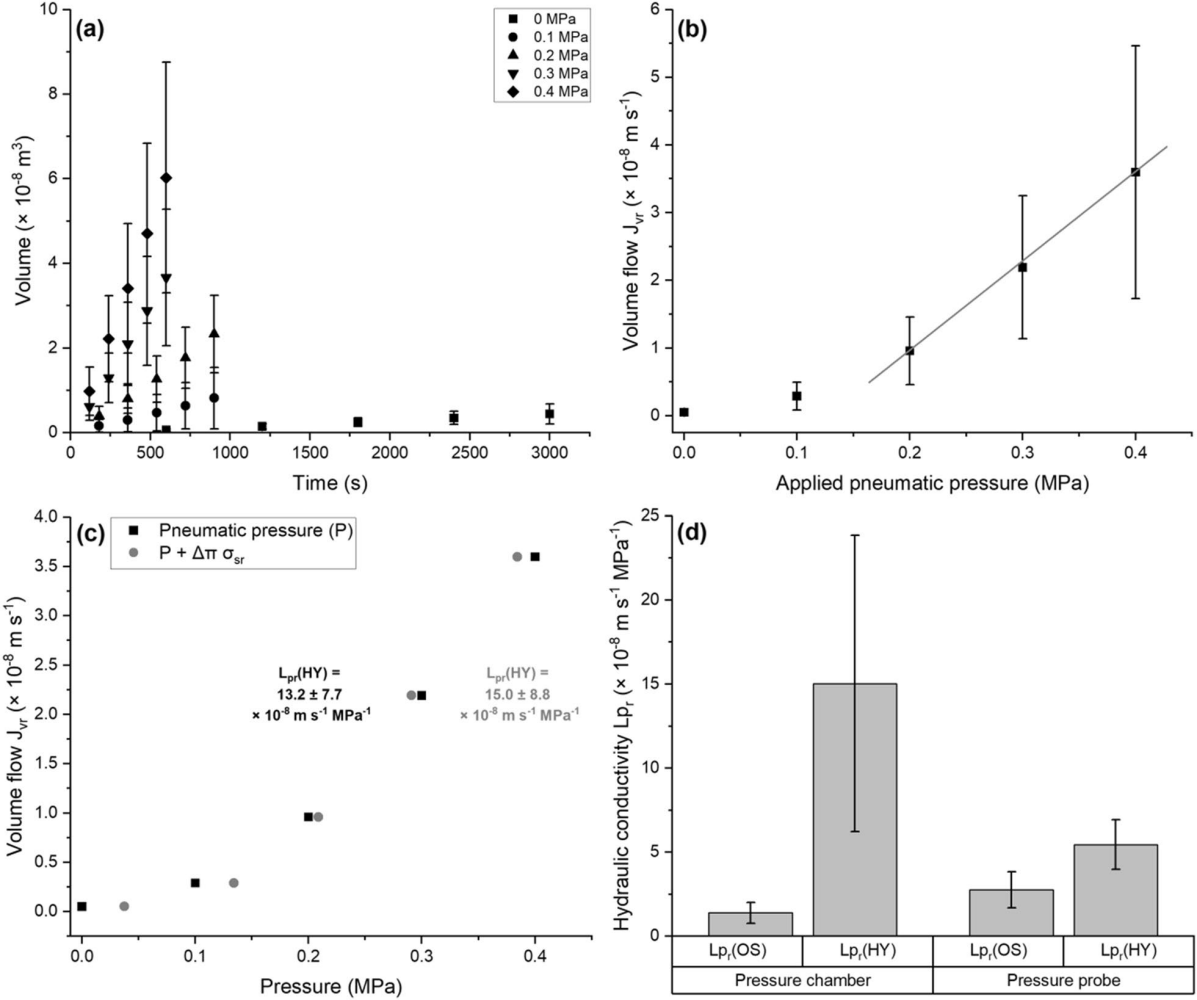
Results of the root transport physiology experiments. Measurements were taken in the absence and presence of applied pneumatic pressure using a pressure chamber (**a**–**d**) or pressure probe (**d**). The roots investigated were cultivated for 5 weeks in hydroponic control conditions. Xylem sap exudates were plotted against the time to allow the calculation of the volume flow (**a**). When the volume flow normalized to the root surface area was plotted as a function of the applied pneumatic pressure (**b**), linear slopes (a line is drawn to guide the eye) were identified in between 0.2 and 0.4 MPa and used to calculate the corresponding hydrostatic hydraulic conductivity Lp_r_(HY). When the volume flow per unit surface area of the root system is drawn as a function of either P or (P + Δπ σ_sr_) (**c**), reduced importance of the osmotic term (Δπ σ_sr_) with higher applied pneumatic pressures (P) due to dilution and filtration effects can be observed. The calculated Lp_r_(HY) is two- to tenfold higher than the osmotic hydraulic conductivity Lp_r_(OS), depending on the method used (**d**). Means with or without standard deviations (n = 10 individual stem cutting root systems or 6 individual whole roots) are shown

The xylem sap exuded from whole root systems increased linearly with time at a given constant pneumatic pressure (Fig. 6a). After normalization of these rates to the root surface area, the volume flow of the root (J_vr_) showed a linear slope at pneumatic pressures between 0.2 to 0.4 MPa (Fig. 6b). To calculate the osmotic hydraulic conductivity (Lp_r_(OS)) the volume flow at 0 MPa was used, whereas the calculation of Lp_r_(HY) was based on the linear slope (0.2 to 0.4 MPa) of each individual experiment. With increasing applied pneumatic pressures (P), the dilution (0 to 0.2 MPa) and filtration (0.2 to 0.4 MPa) of the nutrient solution (see changes in distance of grey to black data points; Fig. 6c) resulted in a decreased importance of the osmotic term (Δπ σ_sr_) as part of the composite driving force (P + Δπ σ_sr_). On average, the hydrostatic hydraulic conductivity (15.0 × 10^–8^ m s^−1^ MPa^−1^) of poplar root systems was tenfold higher than the osmotic hydraulic conductivity (1.4 × 10^–8^ m s^−1^ MPa^−1^) when measured with the pressure chamber (Fig. 6d). In contrast, measurements with the root pressure probe resulted in a twofold higher hydrostatic Lp_r_ (5.5 × 10^–8^ m s^−1^ MPa^−1^) if compared to the osmotic hydraulic conductivity (2.8 × 10^–8^ m s^−1^ MPa^−1^) (Fig. 6d).

### Stress treatments

**Fig. 7:**
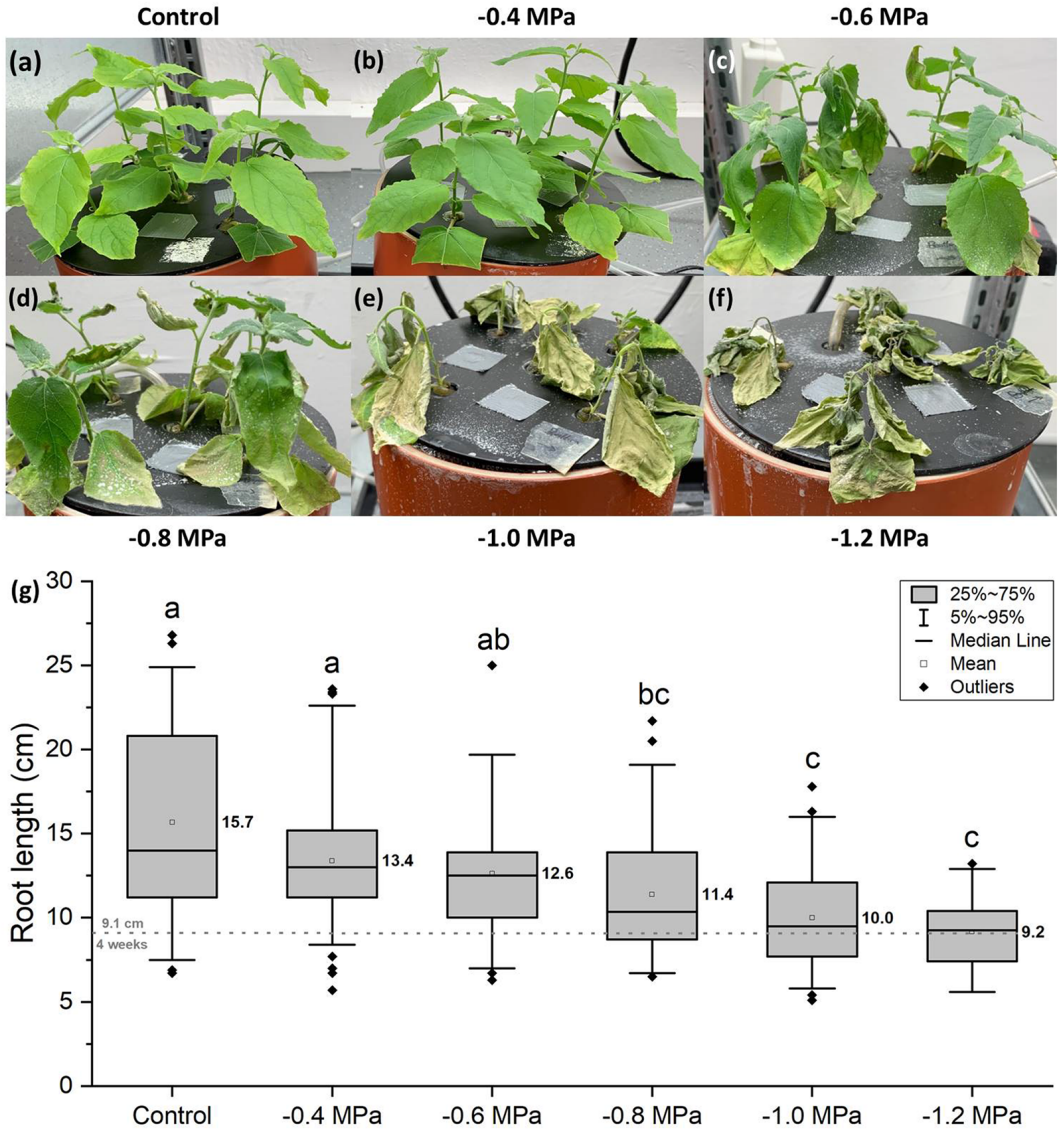
Osmotic stress experimental series. **a**–**f** Pictures visualizing the gradual decline of poplar plant vitality with decreasing osmotic potentials. Osmotic potentials of the nutrient solution below − 0.8 MPa resulted in irreversible plant damage (**e**, **f**). **g** Root lengths after 5 weeks of hydroponic cultivation including a final week of osmotic stress. The dotted reference line for 4-week-old roots is based on Fig. 3c. Roots ≤ 5 cm of length were excluded from the analysis, as they have emerged significantly later and were not entirely exposed to the osmotic stress conditions. Differential letters indicate significant differences (Kruskal–Wallis ANOVA) at p ≤ 0.05. n = 34–81 individual roots from 1 biological replicate per stress treatment

Shortly after the application of the osmotic stress induced by PEG8000, all shoots collapsed for several hours (Additional file 1: Fig. S4a–c). Depending on the severity of the osmotic stress, this collapse was either overcome within 24 h (Fig. 7a–d, Additional file 1: Fig. S4d), or the shoots were never able to recover from the applied osmotic stress (Fig. 7e, f). With decreasing osmotic potentials of the nutrient solution, plant vitality gradually decreased and poplar plants investigated here were unable to cope with osmotic potentials lower than -0.8 MPa (Fig. 7a–f). A similar gradual decline was observed in mean root lengths which were found to be shorter with a decreasing osmotic potential of the nutrient solution (Fig. 7g).

## Discussion

### Plant development in control conditions

Very similar to our observations (Figs. 2, 3a, Additional file 1: Fig. S1), *P. trichocarpa* stem cuttings rooted 6 days after transfer into hydroponics and produced about ten roots per stem cutting on average within 14 days [18]. Based on the root lengths in control conditions (Fig. 3c), 5-week-long hydroponic experiments (Fig. 1) represented the best compromise between plant development, practicality, and comparability especially to previous results produced in our laboratory with barley plants germinated directly from seeds [5, 40, 52]. The time-consuming intermediate step of cultivating tissue culture plants in soil (Fig. 1a) opposed to immediate acclimatization of tissue culture plants to hydroponics [17, 19], offered great advantages. In vitro tissue culture plants are known for severe developmental defects that may be repaired or overcome once the plants are transplanted into less artificial ex vitro growth conditions [61, 62]. Especially illuminated roots are known to produce misleading phenotypic artifacts [63, 64]. The temporary cultivation in soil allowed plants to mature and the stem cuttings to be used in hydroponic experiments. Hydroponics, in turn, enabled several highly important implications for root physiology experiments if compared to tissue culture acclimatization: (i) precisely defined root ages, (ii) possibility of root segmentation into functional developmental zones, (iii) all roots had developed entirely in the dark in the hydroponic system, and (iv) the possibility of applying the root pressure probe or pressure chamber to measure the transport physiology of individual whole roots or intact root systems. For the screening of potential future genetically modified poplar lines and comparison to their corresponding wildtypes, this experimental setup may be imagined as well. It needs to be kept in mind, however, that hydroponic cultivation is still far from natural growth in soil and acquired results need to be interpreted with caution. It may considerably affect the root development, as for example a differential exodermis formation [41, 65], neglects the soil microbiome [17, 66], and measured hydraulic properties are not necessarily indicative of those evaluated with soil-grown plants, which can also be performed using the pressure chamber [67, 68]. Aside from root development, the plants cultivated in hydroponic conditions showed a typical osmotic potential gradient from roots to leaves (Fig. 3b) and an overall proportional development of shoot organs (Figs. 2, 3), also allowing the thorough examination of stress adaptation processes in leaves and stems if desired.

### Histochemical observation of Casparian bands and suberin lamellae in poplar roots

When investigating apoplastic root transport barriers it is of high importance to define certain functional zones. These are typically defined to be a primary endodermal state, where Casparian bands but no suberin lamellae are developed. Here, radial water and nutrient uptake occur via the main root [39]. Chronologically this is followed by a zone of patchy suberization which can be observed in many species [69]. In this zone also lateral roots start to emerge and thus a shift of radial water and nutrient uptake via the main root to its laterals takes place. Lastly, in the zone of full suberization, the primary function of the main root has become the longitudinal transport of water and solutes in the central cylinder to the shoot [70]. In the case of barley, these functional developmental zones were observed to be present at the very same relative distances (0–25%, 25–50%, 50–100%, respectively) even if subjected to osmotic stress [40]. In poplar roots cultivated in control conditions, the first functional zone without suberization was observed between 0 and 27.5%. A full suberization has rarely been observed and the second functional zone was extended up to the root base (27.5–100%) (Fig. 4c). Of course, after exposure to abiotic stress conditions, this functional developmental zonation of poplar roots would also have to be investigated microscopically to verify the relative root zones.

In contrast to cylindrical roots of monocotyledonous crops [36, 40, 71–73], dicotyledonous poplar roots cultivated for 5 weeks in hydroponic conditions showed an increase of root diameter over the length of the root (Fig. 4a) leading to a significantly more complex calculation of the (endodermal) root surface area (cylindrical versus truncated cone geometry). However, if this would not be accounted for, severe over- or underestimations of the true (endodermal) surface area leading to the misinterpretation of relative suberin amounts (Fig. 5) or root hydraulic properties (Fig. 6) may happen. Albeit significant increases in root diameters towards the root base, a final secondary growth and thickening with the development of a periderm [29, 74] was never observed after 5 weeks of cultivation.

Similar to *H. vulgare* [40] and *P.* × *euramericana* [21] but different from *P. deltoides* [22], an exodermis (i.e. hypodermis with Casparian bands; [75]) was never observed with *P.* × *canescens* roots cultivated in hydroponic control conditions. An exodermis in *P.* × *canescens* roots may potentially form in response to abiotic stresses as observed in the roots of monocotyledonous species [46, 52] or in dependence on the cultivation medium as reported for *P. tremuloides* [68, 76].

### Analytical investigation of poplar root suberin

The qualitative composition of the *P.* × *canescens* root suberin (Fig. 5a) including identified functional groups, carbon chain lengths, and most prominent constituents, is highly similar to that of the dicotyledonous *Arabidopsis thaliana* [57, 77], but distinctly different from the bark suberin of the aforementioned *P.* × *canescens* clone INRA 717-1B4 where significantly fewer suberin monomers were identified [78]. Such a deviation of root and bark suberin composition is not surprising, as similar findings were reported for the tree species *Picea abies* previously [79]. The similarity to *A. thaliana* might be explained with comparably close phylogenetic relationships, as the genus of *Populus* has diverged from the *Arabidopsis* lineage about 100 to 120 million years ago [6, 80], which was significantly more recent than the monocot-dicot divergence approximately 200 million years ago [81].

*Populus* is known for its dominant phenolic glycoside and phenylpropanoid metabolism as a defense mechanism [82, 83], which may explain the abundance of benzoic acid derivatives co-solubilized in the suberin analysis (Additional file 1: Fig. S3b, c). In our opinion, it is unlikely yet not impossible that these constituents may partly belong to the aromatic suberin fraction [84]. The chemical analysis of the chloroform:methanol supernatant (Additional file 1: Fig. S3a) revealed significant overlaps with the identified monomers after suberin extraction, particularly of the acids, alcohols, and 2-hydroxy acids functional groups (Fig. 5a). Especially the C16, C18:2, C18:1, and C18 acids are known to be membrane lipid components not associated with the suberin polymer [85]. Therefore, we focused here on the aliphatic suberin diagnostic monomers belonging to the substance classes ω-hydroxy acids and α,ω-dicarboxylic acids. It has been suggested that suberin amounts should best be related to the endodermal surface area, representing an independent parameter, rather than to root dry-weight, which represents a composed parameter [71]. If the total dry-weight of isolated cell wall samples, composed of the cell wall polymers suberin, lignin, and carbohydrates, is increasing, for example only due to lignification, which represents an often observed unspecific response to abiotic stress [86] without changes in amounts of the other polymers, amounts of suberin and carbohydrates will decrease when related to dry-weight, but not when related to the surface area. Even though in poplar roots endodermal surface area, root dry-weight, and root length are increasing proportionally over 5 weeks in control conditions (Fig. 5b–d), relating suberin amounts to the endodermal surface area allows direct comparison to values published for other species. The identified aliphatic suberin amounts (Fig. 5b) are very well comparable to that of 4-week-old *A. thaliana* [57], 30-days-old *Ricinus communis* [33], 10-days-old *Glycine max* [27], 12-days-old *H. vulgare* [39, 40], 12-days-old *Zea mays* [71], and 30- to 40-days-old *Oryza sativa* [71, 87] roots. The comparably high standard deviations of the suberin amounts of poplar roots obtained after chemical analysis (Fig. 5) are probably caused by the generally high variation in poplar root suberization over the root length.

### Root transport physiology

To the best of the author’s knowledge, this is the first study employing the root pressure probe to measure transport properties of poplar roots. If the root hydraulic conductivity of individual whole poplar roots measured with the root pressure probe is compared to excised 5 cm root tip segments (10^–6^ m s^−1^ MPa^−1^) of *P. trichocarpa* × *deltoides* estimated with a vacuum method [88], a significantly higher hydraulic conductivity of the apical root part is observed. Similar findings are reported for roots of barley, which was argued to be caused by increased depositions of suberin lamellae in the basal root part leading to a significantly decreased radial water permeability [39]. Overall, the average Lp_r_ of woody species is often about one order of magnitude smaller than that of herbaceous plants [67, 89]. If compared to barley [40], rice [58], or maize [35] this does not seem to hold true for 5-week-old poplar root systems, which might be correlated to the significantly higher relative growth rates of poplars if compared to other trees in temperate regions [11]. The results indicate that 5-week-old poplar root systems show a transport physiology being intermediate between that of a woody species and herbaceous plants [90]. Some woody plant species are described to show much higher differences (up to three orders of magnitude) between the osmotic and the hydrostatic hydraulic conductivity if compared to herbaceous plants [90]. This tendency, albeit not as large, is also reflected in our measurements revealing a two- to tenfold higher Lp_r_(HY) for poplar roots on average (Fig. 6d). The observable differences in especially Lp_r_(HY) estimated with the root pressure probe or pressure chamber are not surprising and have been reported previously for rice plants cultivated in aeroponics [37]. The investigation of whole root systems rather than individual roots, which in literature very often are only represented by lateral-free apical root tip zones [39, 40], increases the chance of apoplastic bypass generated by damaged or emerging lateral roots [58] which break the apoplastic continuum. However, considering the high variability of root hydraulics, Lp_r_ values measured in the range of 10^–8^ m s^−1^ MPa^−1^ with either method are very well comparable and also in general accordance to those reported for *P. tremuloides* [68, 76, 91]. In addition to the methods presented here, also apoplastic bypass flow experiments with PTS (trisodium, 3-hydroxy-5,8,10-pyrenetrisulfonate) using the pressure chamber [58, 68, 91] can be imagined for future experiments.

### Stress treatments

Poplar plants that were able to recover from the osmotic shock within 24 h (Additional file 1: Fig. S4) were also able to survive the prolonged water deficit treatment of 7 days in our study, resulting in a visible gradient of declining plant vitality with increasing osmotic stress (Fig. 7). Although different poplar species have frequently been investigated in regards to water deficit [68, 76, 92–98] and even osmotic stress using different types of PEG [99–102], such a gradual osmotic stress treatment of the same species has not been reported before. Interestingly, the poplar plants investigated here died with osmotic potentials lower than − 0.8 MPa (Fig. 7), which was only the intermediate osmotic stress intensity (− 0.4, − 0.8, − 1.2 MPa) studied with barley plants in detail [40]. In contrast to poplar, barley plants were able to survive even a water deficit of − 1.2 MPa, indicating a significantly better adaptation to water withdrawal. This increased durability might have evolved as barley originates from more arid regions than poplar trees, which with a few exceptions prefer temperate habitats with high water availability [8, 52, 103].

One of the great benefits of hydroponic cultivation is the precise adjustment of possible abiotic stress treatments, as exemplified in our osmotic stress series (Fig. 7). Other stress conditions might include: salinity [23, 24, 36, 42, 58], exogenous abscisic acid [104, 105], oxygen deficiency [45, 46, 87, 106–108], exposure to heavy metals [21, 22, 25, 38, 41, 65], macronutrient excess or deficiency [33, 43, 44, 109–111], silicon fertilization [5, 112, 113], or even light stress when lightproof plastic pots are replaced by glass beakers. Especially regarding poplars and also the closely related willows (*Salix*), scientific data on root structural and anatomical changes (e.g. alterations in growth rate, root length, or even aerenchyma development) towards environmental stress is available for example for hypoxia [114, 115], heavy metal exposure [21, 22, 116, 117], salinity [118], and osmotic stress [99]. However, suberin deposition has only occasionally been an objective of these investigations. Two studies show that suberin deposition might be beneficial to cope with heavy metal exposure [21, 116], whereas a third study indicates that this increased suberization is not necessarily conserved for all poplar and willow species [22]. As the mentioned studies were based solely on histochemical observations, qualitative analytical approaches in combination with histochemistry, as performed in this study, will be of high value in the future.

## Conclusion

**Fig. 8:**
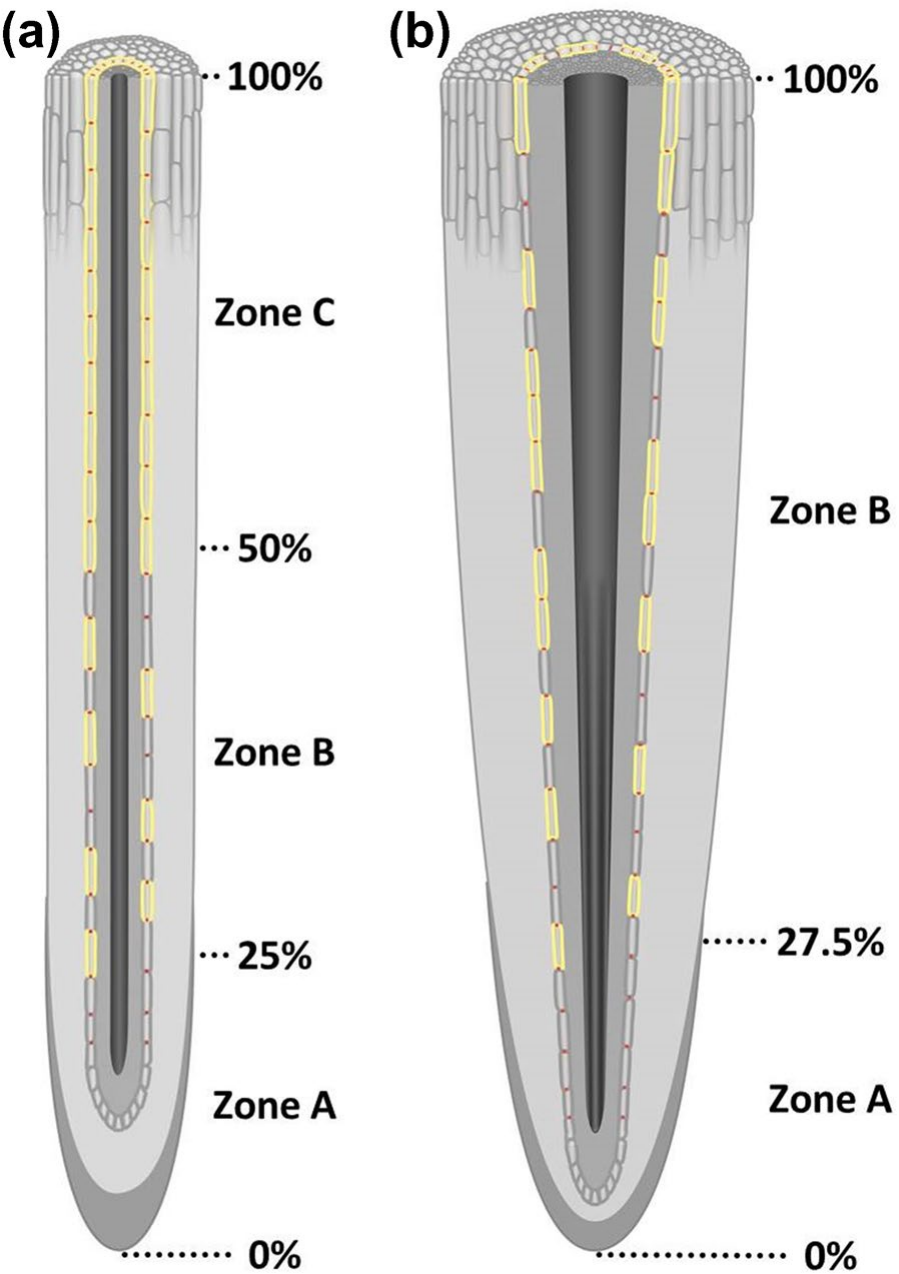
Schematic comparisons between barley and poplar roots cultivated in hydroponic control conditions. Figure **a** is based on data from [40] and the 12-days-old barley plants were cultivated in the same laboratory under identical environmental conditions as the poplar plants grown in hydroponic setups for 5 weeks (**b**). In the apical zone A (0–25%; 0–27.5% relative root length), only Casparian bands and no suberin lamellae were developed. Zone B (25–50%; 27.5–100%) was characterized by a patchy suberization of the endodermis. In contrast to barley seminal roots (50–100%), no basal zone C exhibiting full suberization of the endodermis could be identified in poplar adventitious roots. For simplification, only main roots without lateral roots are shown. Red dots indicate Casparian bands, yellow lines indicate suberin lamellae

We described the set-up of a hydroponics pipeline for *P.* × *canescens* cultivation, which allows detailed and reproducible studies of root anatomy, suberization, and transport physiology. These cultivation conditions also allow a precise developmental comparison, even between cylindrical roots of monocotyledonous plants and conical roots of dicotyledonous plant species showing a continuous increase in thickness over the root length (Fig. 8).

## Supplementary Information

**Additional file 1: Figure S1.** Daily monitoring of the stem cutting development during the rooting phase. Swelling of buds, bud break, first root emergence, and relative rooting efficiency were monitored daily. To calculate the relative rooting efficiency, all rooted stem cuttings were divided by the number of prepared stem cuttings of a given PVC disk in the rooting phase. Boxplots are based on n = 54 independent PVC disks. **Figure S2.** Developmental state of poplar leaves after 5 weeks of hydroponic cultivation in control conditions. Chlorophyll content (a), combined projected surface area (b), and stomatal conductance (c) of leaves were analyzed to characterize the leaf development during hydroponic cultivation. Boxplots are based on n = 35 (a), 26 (c) leaves, and 30 (b) shoots. **Figure S3.** Chemical analysis of compounds released before or during poplar root suberin analysis. (a) Chromatogram of the chloroform:methanol extracts obtained from enzymatically isolated suberized cell walls. i.s. = internal standard, acid = primary acid, alcohol = primary alcohol, 2-OH acid = 2-hydroxy acid, ω-OH acid = ω-hydroxy acid. (b, c) Benzaldehyde and benzoic acid derivatives in suberin extracts from poplar roots. Roots were harvested after 5 weeks of hydroponic cultivation in control conditions. The representative qualitative and quantitative composition of zone B is given (b). OH = hydroxyl. Benzaldehyde and benzoic acid derivatives are defined by additional hydroxyl groups at various positions of the aromatic ring structure (c). Means with standard deviations (n = 10 biological replicates) are shown. **Figure S4.** Pictures visualizing the collapse of poplar shoots shortly after osmotic stress application. If plants were able to cope with the applied osmotic stress, the shoots fully recovered within 24 h (d). Representative pictures of the -0.6 MPa treatment are shown.

## Data Availability

The datasets used and/or analyzed during the current study are available from the corresponding author on reasonable request. All data generated or analyzed during this study are included in this published article and its Additional files.
