# Supplementary figures and images for: Hydroponic cultivation conditions allowing the reproducible investigation of poplar root suberization and water transport
Source: Plant Methods. 2021 Dec 15;17:129. doi: 10.1186/s13007-021-00831-5 (PMC8672600; doi:10.1186/s13007-021-00831-5)

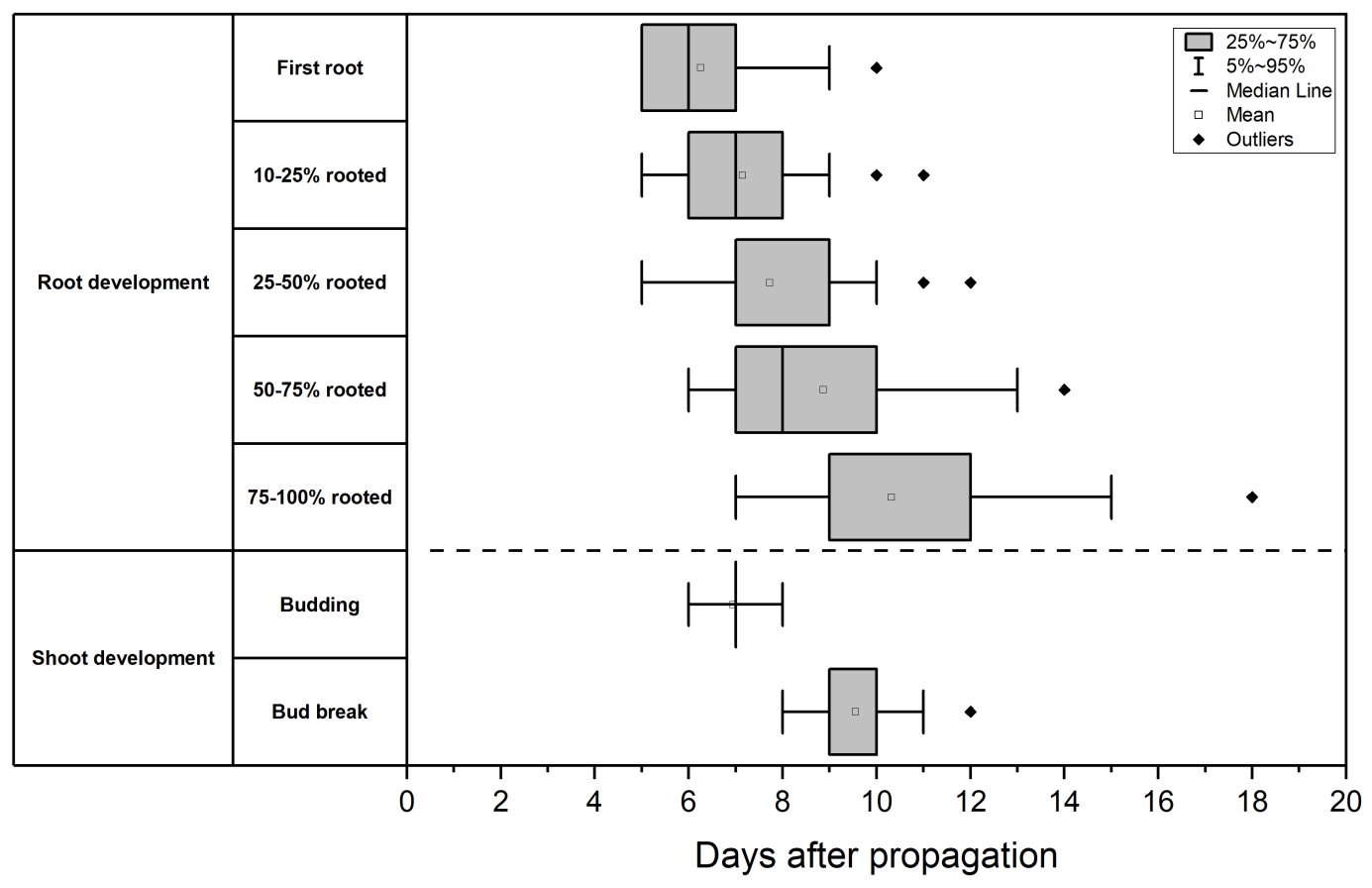


Figure S1


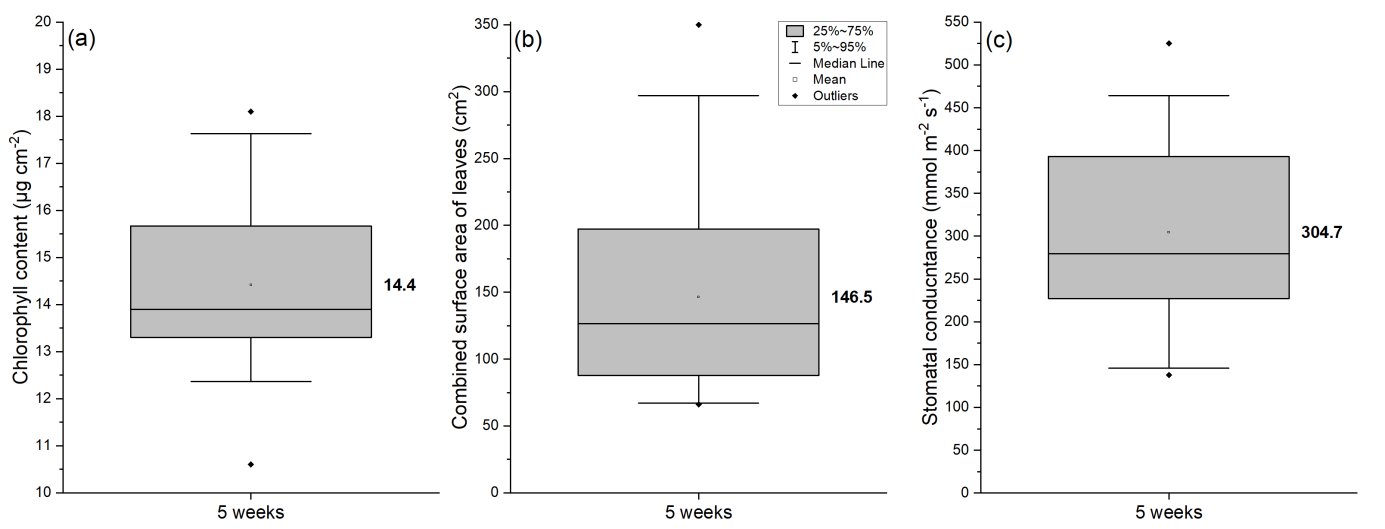


Figure S2


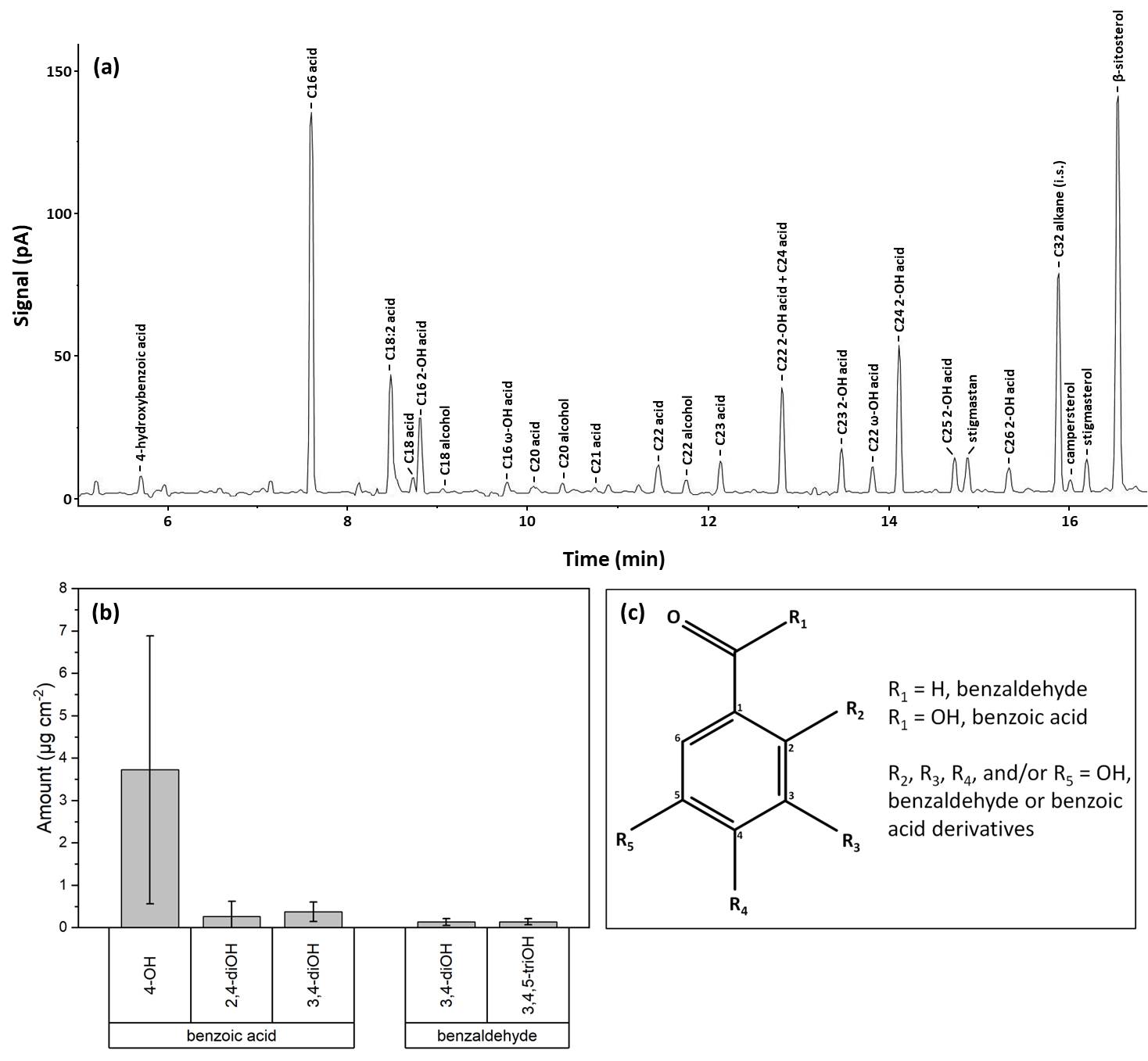


Figure S3


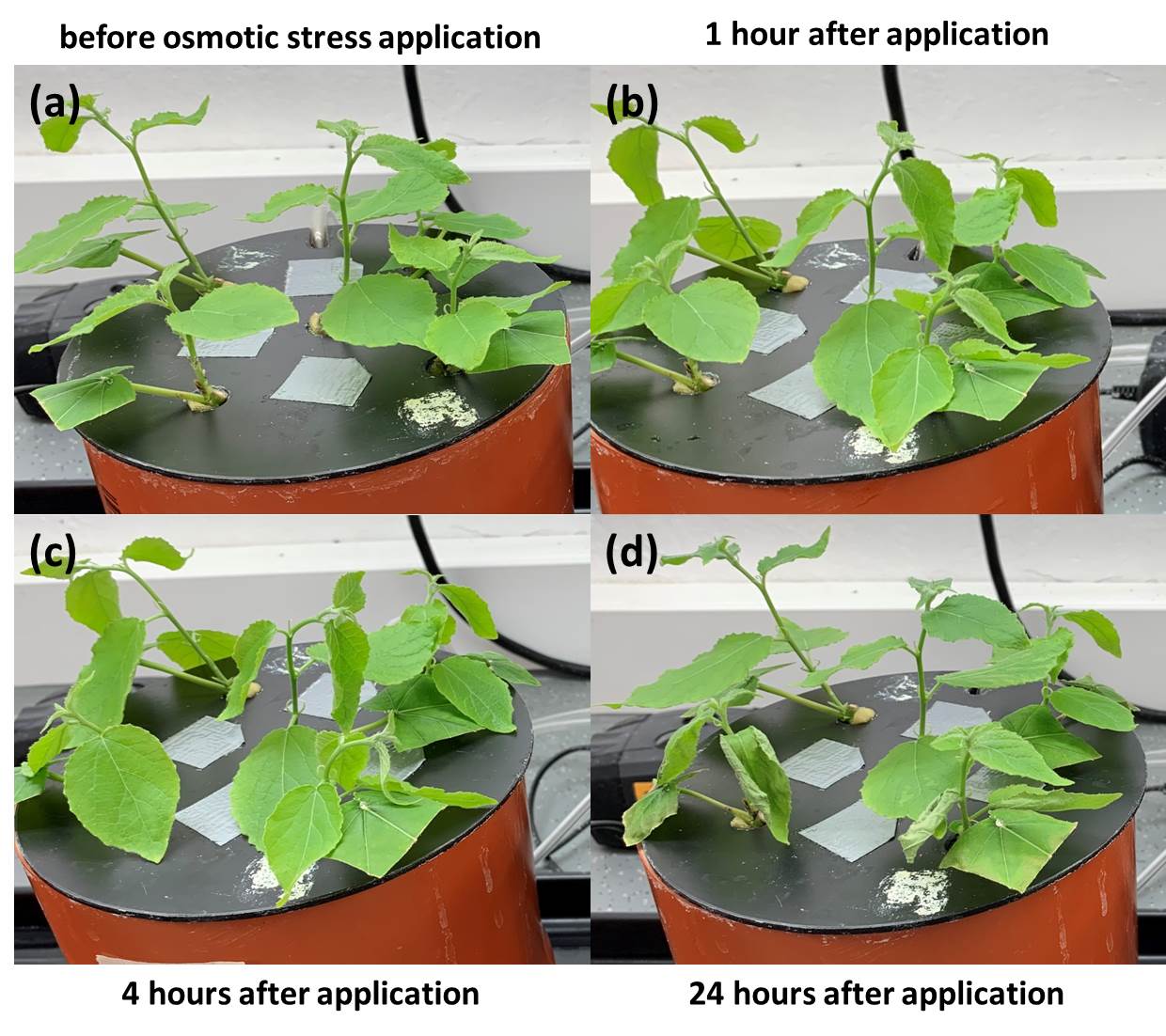


Figure S4

Supplement: Supplementary file 1 — Additional file 1: Figure S1. Daily monitoring of the stem cutting development during the rooting phase. Swelling of buds, bud break, first root emergence, and relative rooting efficiency were monitored daily. To calculate the relative rooting efficiency, all rooted stem cuttings were divided by the number of prepared stem cuttings of a given PVC disk in the rooting phase. Boxplots are based on n = 54 independent PVC disks. Figure S2. Developmental state of poplar leaves after 5 weeks of hydroponic cultivation in control conditions. Chlorophyll content (a), combined projected surface area (b), and stomatal conductance (c) of leaves were analyzed to characterize the leaf development during hydroponic cultivation. Boxplots are based on n = 35 (a), 26 (c) leaves, and 30 (b) shoots. Figure S3. Chemical analysis of compounds released before or during poplar root suberin analysis. (a) Chromatogram of the chloroform:methanol extracts obtained from enzymatically isolated suberized cell walls. i.s. = internal standard, acid = primary acid, alcohol = primary alcohol, 2-OH acid = 2-hydroxy acid, ω-OH acid = ω-hydroxy acid. (b, c) Benzaldehyde and benzoic acid derivatives in suberin extracts from poplar roots. Roots were harvested after 5 weeks of hydroponic cultivation in control conditions. The representative qualitative and quantitative composition of zone B is given (b). OH = hydroxyl. Benzaldehyde and benzoic acid derivatives are defined by additional hydroxyl groups at various positions of the aromatic ring structure (c). Means with standard deviations (n = 10 biological replicates) are shown. Figure S4. Pictures visualizing the collapse of poplar shoots shortly after osmotic stress application. If plants were able to cope with the applied osmotic stress, the shoots fully recovered within 24 h (d). Representative pictures of the -0.6 MPa treatment are shown. [file 13007_2021_831_MOESM1_ESM.docx]
